# Anosognosia in Amnestic Mild Cognitive Impairment Is Related to Diminished Hippocampal Volume Comparable to Alzheimer’s Disease Dementia: Preliminary MRI Findings

**DOI:** 10.3389/fnagi.2021.739422

**Published:** 2021-10-28

**Authors:** Juan Francisco Flores-Vázquez, Gabriel Ramírez-García, Oscar René Marrufo-Meléndez, Ruth Alcalá-Lozano, Morten Peter Lietz, Yaneth Rodríguez-Agudelo, Gilberto Isaac Acosta-Castillo, Remco J. Renken, Andre Aleman, Stefanie Enriquez-Geppert, Ana Luisa Sosa-Ortiz

**Affiliations:** ^1^Programa de Maestría y Doctorado en Ciencias Médicas, Odontológicas y de la Salud, Universidad Nacional Autónoma de México, Mexico City, Mexico; ^2^Laboratorio de Demencias, Instituto Nacional de Neurología y Neurocirugía Manuel Velasco Suárez, Mexico City, Mexico; ^3^Department of Clinical and Developmental Neuropsychology, University of Groningen, Groningen, Netherlands; ^4^Section of Cognitive Neuropsychiatry, Department of Biomedical Sciences of Cells and Systems, University of Groningen, Groningen, Netherlands; ^5^Laboratorio de Neuropsicología, Departamento de Fisiología, Facultad de Medicina, Universidad Nacional Autónoma de México, Mexico City, Mexico; ^6^Unidad de Neuroimagen, Instituto Nacional de Neurología y Neurocirugía Manuel Velasco Suárez, Mexico City, Mexico; ^7^Subdirección de Investigaciones Clínicas, Instituto Nacional de Psiquiatría Ramón de la Fuente Muñiz, Mexico City, Mexico; ^8^Departamento de Neuropsicología, Instituto Nacional de Neurología y Neurocirugía Manuel Velasco Suárez, Mexico City, Mexico

**Keywords:** mild cognitive impairment, Alzheimer’s disease, anosognosia, hippocampus, magnetic resonance imaging

## Abstract

Although the presence of anosognosia in amnestic mild cognitive impairment (aMCI) may be predictive of conversion to Alzheimer’s disease (AD), little is known about its neural correlates in AD and aMCI. Four different groups were compared using volumetric and diffusion magnetic resonance imaging metrics in regions of interest (hippocampus and cingulum cortex gray matter, cingulum bundle white matter): aMCI subjects with anosognosia (*n* = 6), aMCI subjects without anosognosia (*n* = 12), AD subjects with anosognosia (*n* = 6), and AD subjects without anosognosia (*n* = 9). aMCI subjects with anosognosia displayed a significantly lower gray matter density (GMD) in the bilateral hippocampus than aMCI subjects without anosognosia, which was accounted for by bilateral hippocampal differences. Furthermore, we identified that the mean hippocampal gray matter density of aMCI subjects with anosognosia was not statistically different than that of AD subjects. The groups of aMCI and AD subjects with anosognosia also displayed a lower GMD in the bilateral cingulum cortex compared to subjects without anosognosia, but these differences were not statistically significant. No statistically significant differences were found in the fractional anisotropy or mean diffusivity of the hippocampus or cingulum between subjects with and without anosognosia in aMCI or AD groups. While these findings are derived from a small population of subjects and are in need of replication, they suggest that anosognosia in aMCI might be a useful clinical marker to suspect brain changes associated with AD neuropathology.

## Introduction

Anosognosia is defined as the loss or decline in a subject’s awareness of problems in daily functioning, behavior, cognition, or mood (Starkstein, 2014). This condition is frequent throughout the trajectory of Alzheimer’s disease (AD), with a prevalence ranging between 20 and 80%, and its presence is linked to increased dependence, reduced adherence to treatment and risk behaviors in patients, increased caregiver distress, and a greater economic burden for families and societies (Turró-Garriga et al., 2013; Starkstein, 2014).

Anosognosia can appear in amnestic mild cognitive impairment (aMCI), a diagnosis that implies a heightened risk for developing AD dementia, in which memory performance is diminished but autonomy in daily life is preserved (Mak et al., 2015). Interestingly, the presence of anosognosia in aMCI has been associated with underlying brain changes characteristic of AD, and may have a predictive value for further worsening of cognition and progression to AD dementia (Scherling et al., 2016; Gerretsen et al., 2017; Therriault et al., 2018; Hanseeuw et al., 2020). As only a fraction of aMCI-affected subjects will progress to dementia, the clinical characterization of subjects at a higher risk of developing AD is a major concern in current research. This, in turn, can support timely interventions before significant neural and functional impairment has taken place. The understanding of the neural changes associated with anosognosia is therefore necessary for the adequate characterization of aMCI and the early stages of AD (Mondragón et al., 2021).

According to a recent systematic review, anosognosia in AD is associated with a reduction in gray matter density, cerebral blood flow, and metabolism in several regions: the anterior and posterior cingulate cortex, the medial temporal lobe, the inferior, superior and medial frontal gyri, the orbitofrontal cortex, and the insula (Hallam et al., 2020). Most of these regions form part of the default mode network, a large-scale brain network that is altered from the early stages of the AD continuum (Grieder et al., 2018) and is associated with self-related cognition (e.g., introspection and autobiographic memory) (Zamboni et al., 2013). Said systematic review identified that measurement heterogeneity is one of the main limitations in integrating previous clinical studies on anosognosia in aMCI and AD dementia.

In clinical research, three methods have been mainly used to measure anosognosia in AD (Hallam et al., 2020). (1) *Clinical rating*, in which the clinician’s judgment is used to rate the level of anosognosia on a scale after an interview with the patient and the caregiver. (2) *Patient-informant discrepancy*, in which after parallel interviewing of the patient and caregiver, a “discrepancy score” of the patient’s symptoms is calculated, and finally. (3) *Performance discrepancy*, in which the performance of a patient on a neuropsychological test is compared to their own estimation of how well they think they performed on said test. Taking this into consideration, a multi-method magnetic resonance imaging (MRI) morphometric study showed that all three anosognosia measurement methods were independently associated with gray matter atrophy in the medial temporal lobe including the right hippocampus in AD participants (Tondelli et al., 2018). The consistent involvement of the medial temporal lobe and the hippocampus supports the view that anosognosia is principally caused by a decline in memory processes (such as the autobiographical episodic memory loss typically characterizing AD) that prevents the update of self-knowledge (Morris and Mograbi, 2013; Chavoix and Insausti, 2017). However, the neural substrate of anosognosia in AD is far from being fully elucidated, and there is a need for replication of previous findings along with the development of objective anosognosia measurements (Hallam et al., 2020).

A sound approach for the assessment of anosognosia has been developed under the more general construct of the behavioral dysexecutive syndrome (Godefroy et al., 2010). This syndrome groups twelve symptoms related to disturbances in the executive function brain network (e.g., anosognosia, apathy, irritability, and confabulations) that are frequently observed in several neurocognitive disorders. In mild AD dementia, 86% of patients have been found to exhibit a behavioral dysexecutive syndrome, with a large effect size when comparing the severity and of anosognosia between AD participants and healthy controls (Godefroy et al., 2014). Along with the definition of the syndrome, a straightforward structured questionnaire has been proposed, the Behavioral Dysexecutive Syndrome Inventory (BDSI), which is applied to an informant who knows the patient well, and aims to measure the frequency and severity of each symptom – including anosognosia (Godefroy et al., 2010).

In light of this evidence, we aimed to provide further evidence on the neural underpinnings of anosognosia in aMCI and AD using the BDSI in a clinical sample. To our knowledge, no previous studies have used this anosognosia measurement to study structural brain changes. We hypothesized that subjects with aMCI or AD dementia with anosognosia would exhibit volumetric and white-matter integrity changes in the bilateral hippocampus and cingulate cortex, relative to aMCI or AD subjects without anosognosia.

## Materials and Methods

### Sample and Participants

Eighteen participants with aMCI and sixteen participants with AD were recruited from the outpatient consultation of the Dementia Laboratory of the National Institute of Neurology and Neurosurgery of Mexico in Mexico City. aMCI and AD dementia were diagnosed by certified specialists following current clinical criteria (Petersen, 2004; McKhann et al., 2011). Inclusion criteria further consisted of: being between 60 and 76 years of age, a Mini-Mental Score Examination (MMSE) score of 25 or higher in the aMCI group and 16 or higher in the AD dementia group, and having a knowledgeable informant living with the subject who could answer to the clinical questionnaires. Exclusion criteria consisted of a clinical history suggestive of non-AD dementia, current symptoms suggestive of delirium, major depression, substance-use disorders, or other major neuropsychiatric disorders (apart from aMCI or AD dementia), not being able to complete clinical or neuroimaging assessments, and MRI contraindications.

### Clinical Measurements

General cognitive functioning was assessed using an adapted version of the MMSE widely used in Mexico (Ostrosky-Solís et al., 2000). For the assessment of anosognosia, a cross-culturally adapted Mexican version (Flores-Vazquez and Sosa-Ortiz, 2016) of the BDSI (Godefroy et al., 2010) was used. Subjects were divided into “anosognosia” or “no-anosognosia” groups if the answer given to the screening questions presented in Table 1 was “yes” or “no.” Additional analysis taking into consideration the severity and frequency of anosognosia can be consulted in the Supplementary Material.

**TABLE 1:** Anosognosia assessment in the behavioral dysexecutive syndrome inventory (Godefroy et al., 2010).

**Screening questions:** Does the subject minimize or fail to recognize the limitations or difficulties that they have and the consequences of those limitations in daily life? Does the subject make unrealistic plans? Does the subject think that they can do the same things they used to do even when this is no longer realistic?

**Specific questions** (used for clarification): • Does the subject tend to minimize their own cognitive decline, for instance, their memory problems? • Does the subject tend to minimize their behavioral problems? • Does the subject tend to minimize their impairments when moving, seeing, or hearing? • Is the subject indifferent to their impairments although these impairments impact their daily life? • Does the subject blame their impairments on other people or the situation? • Does the subject deny their impairments although other people can notice them? • Does the subject act as if they had no illness and need no help from others? • Does the subject make unrealistic plans and wrongly thinks they could retake previous activities?
**Frequency scoring:** 1 = Rarely: less than once a week. 2 = Sometimes = approximately once a week. 3 = Frequently: several times a week, but not every day. 4 = Very frequently = every day/most of the time.
**Severity scoring:** 1 = Mild: noticeable, few consequences in everyday life. 2 = Moderate: significant and disturbing, but manageable. 3 = Severe: very marked and disturbing, very difficult to manage
^*a*^ If the answer to any of the screening questions is “yes,” the anosognosia domain should be examined at depth using the specific questions, and the frequency and severity of the anosognosia domain should be scored

### Magnetic Resonance Imaging Acquisition and Processing

Magnetic resonance imagings were acquired using a 3 Tesla SIEMENS Skyra scanner (Erlangen, Germany) with a 20ch head coil.

**FIGURE 1:**
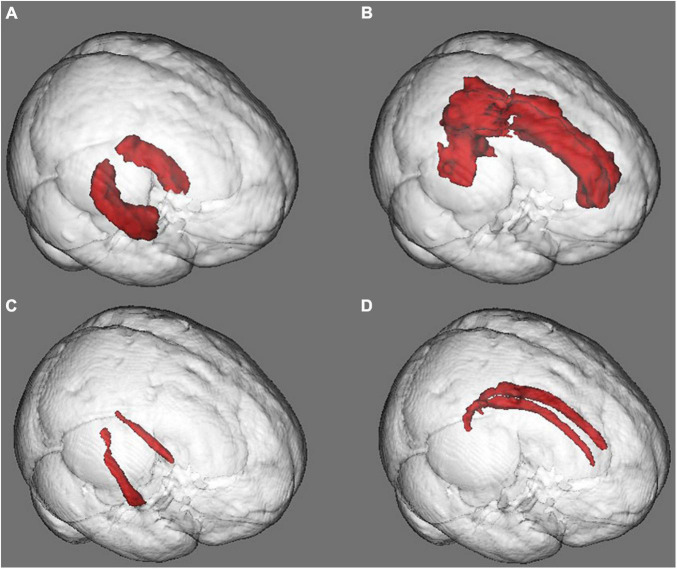
Gray and white matter regions of interest (ROIs). **(A)** Hippocampal gray matter ROI. **(B)** Cingulum gray matter ROI. **(C)** Hippocampal white matter ROI. **(D)** Cingulum white matter ROI. The morphological or diffusion values were averaged in each ROI for between-group comparisons.

T1-weighted images were obtained using a 3D MPRAGE sequence (TR/TE: 2,300/2.45 ms; FOV: 256 mm^2^; matrix: 256 × 256; voxel size: 1 mm^3^). Preprocessing included denoising and intensity inhomogeneity correction (Manjón et al., 2010; Avants et al., 2011). T1-weighted images were processed using the VBM-FSL toolbox (Douaud et al., 2007; Jenkinson et al., 2012). T1 image processing included brain extraction, tissue-type segmentation, the creation of a study-specific gray matter template, registration of all gray matter images into the template, Jacobian modulation, and smoothing. Of note, including the Jacobian modulation step in the processing pipeline handles the variability in the head size of the subjects at a local level, eliminating the need for controlling or correcting for by ICV (Douaud et al., 2007). The gray-matter regions of interest (ROIs) of the bilateral hippocampus and cingulate cortex were defined using the Harvard-Oxford Cortical and Subcortical atlases (Desikan et al., 2006), respectively (Figure 1). First, each ROI was eroded to reduce its size according to the anatomical region into the MNI standard space; then, all ROIs were binarized. These ROIs were used to extract the mean gray matter density (GMD).

Diffusion-weighted images (DWI) were obtained using an echo-planar spin-echo sequence with 64 directions (TR/TE: 5,000/102 ms; FOV: 220 mm^2^; matrix: 100 × 100; voxel size: 2.2 mm^3^). DWI image preprocessing included a first denoising step using a blockwise non-local means filter (Coupé et al., 2008), after which, eddy current and subject movement correction, binary mask creation, and diffusion tensor fitting to obtain the scalar anisotropy and diffusivity maps were completed using the TBSS-FSL toolbox (Smith et al., 2006; Jenkinson et al., 2012). Fractional anisotropy (FA) and mean diffusivity (MD) maps were non-linearly registered to the MNI standard space following the TBSS steps (Smith et al., 2006; Jenkinson et al., 2012). The white-matter ROIs of the bilateral cingulum bundle (Figure 1) were defined using the JHU-DTI-81 White-Matter Labels atlas (Wakana et al., 2007). First, each ROI was eroded to reduce its size according to the anatomical region into the MNI standard space; then, all ROIs were binarized. These ROIs were used to extract the mean FA or MD of the specific white matter tracts.

### Statistical Analysis

Results are presented in means (M), medians (Mdn), interquartile ranges (IQR), and value ranges (Rng). For the description and comparison of demographic and clinical characteristics, Mann-Whitney *U* tests were used for analyzing continuous variables (years age, intracranial volume – ICV, and MMSE score), and Chi-square tests for dichotomous variables (sex).

For the group comparisons (aMCI-anosognosia vs. aMCI-no-anosognosia; AD-anosognosia vs. AD-no-anosognosia) of the averaged GMD or white matter FA in each ROI, Mann-Whitney *U* tests were used. An alpha of 0.05 was defined for statistically significant findings. In the case of statistically significant findings, we conducted a False Discovery Rate analysis, and present the Benjamini-Hochberg adjusted *p* value. The rank-biserial correlation (*r*_*rb*_) is reported as a non-parametric effect size for the Mann-Whitney *U* test (Kerby, 2014). Statistical analyses were carried out in the JASP software version 0.14.1 (JASP Team, 2020) and IBM SPSS Statistics version 27 (IBM Corp, 2020).

Additional analysis assessing the correlation of the severity and frequency of anosognosia with the MRI metrics can be consulted in the Supplementary Material.

### Ethical Considerations

This study was approved by the research and ethics committees of the National Institute of Neurology and Neurosurgery of Mexico after independent, blinded review (protocol number: 116/16) and carried out according to the Declaration of Helsinki. All participating subjects, as well as first-grade family members in the case of subjects with AD dementia, were informed about the study in detail and consented to it.

## Results

### Demographic and Clinical Characteristics

In the aMCI group (*n* = 18), subjects displaying anosognosia (*n* = 6 and 2 female) had a Mdn of 72.0 years of age (*IQR* = 3.0) and a Mdn of 12.0 years of education (*IQR* = 5.3), obtained a Mdn MMSE score of 26.5 (*IQR* = 1.8), and had a Mdn ICV of 1358.4 (*IQR* = 147.0) cm^3^. aMCI subjects not displaying anosognosia (*n* = 12 and 9 female) had a Mdn of 69.5 years of age (*IQR* = 7.0) and a Mdn of 12.0 years of education (*IQR* = 7.0), obtained a Mdn score of 28.0 in the MMSE (*IQR* = 1.3), and had a Mdn ICV of 1292.0 cm^3^ (*IQR* = 64.5). Between-group differences were not statistically significant in any of these variables, median, ranges, test statistics, and *p* values are presented in Table 2.

**TABLE 2:** Demographic and clinical characteristics of patients with aMCI and AD displaying and not displaying anosognosia.

	**aMCI (*n* = 18)**			
	**Anosognosia** **(*n* = 6)**	**No anosognosia** **(*n* = 12)**	**Test statistic**	***p* value**
**Age** (Years)	*M* = 70.7 *Mdn* = 72.0 *IQR* = 3.0 *Rng* = 62.0 – 74.0	*M* = 68.8 *Mdn* = 69.5 *IQR* = 7.0 *Rng* = 60.0–75.0	27.5	0.45
**Sex** (Female, male)	2, 4	9, 3	2.9	0.09
**Education** (Years)	*M* = 12.8 *Mdn* = 12.0 *IQR* = 5.3 *Rng* = 6.0 – 17.0	*M* = 11.9 *Mdn* = 12.0 *IQR* = 7.0 *Rng* = 9.0 – 17.0	29.5	0.57
**MMSE** (Score)	*M* = 26.3 *Mdn* = 26.5 *IQR* = 1.8 *Rng* = 25.0 – 28.0	*M* = 27.6 *Mdn* = 28.0 *IQR* = 1.3 *Rng* = 25.0 – 30.0	53.5	0.10
**ICV** (cm^3^)	*M* = 1312.1 *Mdn* = 1358.4 *IQR* = 147.0 *Rng* = 1058.9 – 1466.8	*M* = 1272.4 *Mdn* = 1292.0 *IQR* = 64.5 *Rng* = 1164.8.0 – 1397.2	25.0	0.34

	**AD (*n* = 16)**			
	**Anosognosia** **(*n* = 7)**	**No anosognosia** **(*n* = 9)**	**Test statistic**	***p* value**

**Age** (Years)	*M* = 71.6 *Mdn* = 72.0 *IQR* = 6.0 *Rng* = 65.0–76.0	*M* = 66.1 *Mdn* = 64.5 *IQR* = 6.0 *Rng* = 60.0–76.0	14.0	0.07
**Sex** (Female, male)	5, 2	5, 4	0.4	0.52
**Education** (Years)	*M* = 11.4 *Mdn* = 9.0 *IQR* = 3.5 *Rng* = 9.0–19.0	*M* = 12.6 *Mdn* = 12.0 *IQR* = 7.0 *Rng* = 6.0–20.0	38.5	0.47
**MMSE** (Score)	*M* = 21.3 *Mdn* = 21.0 *IQR* = 4.0 *Rng* = 17.0 - 26.0	*M* = 23.3 *Mdn* = 22.0 *IQR* = 4.0 *Rng* = 18.0 - 30.0	43.5	0.26
**ICV** (cm^3^)	*M* = 1379.1 *Mdn* = 1358.1 *IQR* = 137.6 *Rng* = 1184.1–1414.8	*M* = 1284.5 *Mdn* = 1293.4 *IQR* = 117.3 *Rng* = 1262.6–1519.2	13.0	0.06

*Values are presented as means (M), medians (Mdn), interquartile ranges (IQR) and ranges (Rng). Test statistics and *p* values for age, education, MMSE and ICV correspond to the Mann-Whitney *U* test. Test statistic and *p* value for sex corresponds to the Chi-square test. aMCI, Amnestic mild cognitive impairment; AD, Alzheimer’s disease dementia; MMSE, Mini-Mental State Examination; ICV, Intracranial volume.*

In the AD group (*n* = 16), subjects displaying anosognosia (*n* = 7 and 5 female) had a Mdn of 72.0 years of age (*IQR* = 6.0), and a Mdn of 9.0 years of education (*IQR* = 3.5), obtained a Mdn score of 21.0 in the MMSE (*IQR* = 4.0), and had a Mdn ICV of 1358.1 cm^3^ (*IQR* = 137.6). AD subjects not displaying anosognosia (*n* = 9 and 5 female) had a Mdn of 64.5 years of age (*IQR* = 6.0) and a Mdn of 12 years of education (*IQR* = 7.0), obtained a Mdn score of 22.0 in the MMSE (*IQR* = 4.0), and had a Mdn ICV of 1293.4 cm^3^ (*IQR* = 117.3). Between-group differences were not statistically significant, and mean, ranges, test statistics with *p* values are presented in Table 2.

### Gray Matter Volumetric Comparisons

**FIGURE 2:**
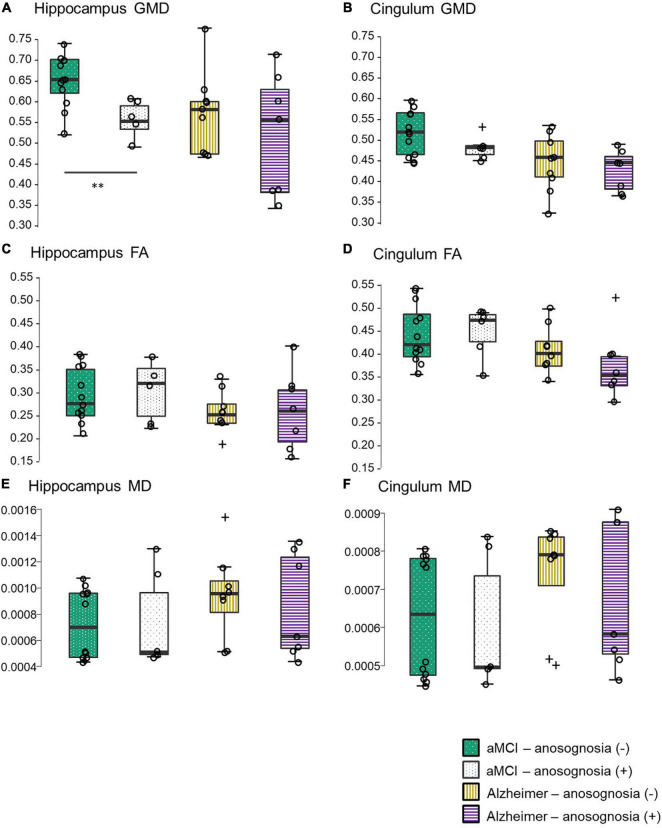
Gray matter density in the hippocampal and cingulum regions of interest in aMCI subjects with and without anosognosia, and AD dementia participants. aMCI subjects with anosognosia displayed significantly less hippocampal gray matter density than aMCI subjects without anosognosia (**p = 0.01). The hippocampal gray matter density of aMCI subjects with anosognosia was not statistically different from participants with AD dementia. Boxplots represent mean group values and interquartile ranges, outliers (values outside the lower of upper limits of the quartile range) are represented with the symbol +. GMD, gray matter density; FA, fractional anisotropy; MD, mean diffusivity; aMCI, amnestic mild cognitive impairment; AD, Alzheimer’s disease. Although most of these correlations were not significant, there are trends shown in Supplementary Figure 1 that could inform future research, particularly the negative correlation between anosognosia scores and FA in both ROIs shown in AD participants. If replicated, this suggests that white-matter integrity disturbances could result in a more pronounced anosognosia presentation in AD.

In the aMCI group, subjects with anosognosia had a lower GMD in the bilateral hippocampus ROI (*M* = 0.55, *Mdn* = 0.54, *IQR* = 0.06, and *Rng* = 0.48–0.60) than subjects without anosognosia (*M* = 0.64, *Mdn* = 0.65, *IQR* = 0.08, and *Rng* = 0.51–0.73). This difference was statistically significant, showing a large effect size (*W* = 63.0, *p* = 0.01, *adjusted p* = 0.04, and *r*_*rb*_ = 0.75, see Figure 2), and was accounted for by bilateral hippocampal differences (right hippocampus: *anosognosia group M* = 0.55, *Mdn* = 0.54, *IQR* = 0.07, and *Rng* = 0.45–0.62; *non-anosognosia group M* = 0.67, *Mdn* = 0.67 *IQR* = 0.12, *Rng* = 0.55–0.77, *W* = 64.0, *p* = 0.01, and *r*_*rb*_ = 0.78; left hippocampus: *anosognosia group M* = 0.55, *Mdn* = 0.56, *IQR* = 0.05, and *Rng* = 0.51–0.57; *non-anosognosia group M* = 0.62, *Mdn* = 0.63, *IQR* = 0.06, *Rng* = 0.48–0.69 *W* = 62.0, *p* = 0.01, and *r*_*rb*_ = 0.72).

The group of aMCI subjects with anosognosia also displayed a lower GMD in the bilateral cingulum cortex ROI (*M* = 0.47, *Mdn* = 0.47, *IQR* = 0.02, and *Rng* = 0.44–0.51) than subjects without anosognosia (*M* = 0.51, *Mdn* = 0.51, *IQR* = 0.11, and *Rng* = 0.43–0.60), but this difference was not statistically significant (*W* = 49.0, *p* = 0.25, and *r*_*rb*_ = 0.36).

In the AD group, subjects with anosognosia had a lower GMD in the bilateral hippocampus ROI (*M* = 5.1, *Mdn* = 0.55, *IQR* = 0.25, and *Rng* = 0.33–0.71) than aMCI subjects without anosognosia (*M* = 0.56, *Mdn* = 0.57, *IQR* = 0.13, and *Rng* = 0.46–0.77), but this difference was not statistically significant (*W* = 39.0, *p* = 0.47, and *r*_*rb*_ = 0.24). In an exploratory analysis, we identified that the Mdn hippocampal GMD of aMCI subjects with anosognosia (*M* = 0.55, *Mdn* = 0.54, *IQR* = 0.06, and *Rng* = 0.48–0.60) was not statistically different than that the whole subset of AD subjects (*M* = 0.54, *Mdn* = 0.56 *IQR* = 0.14, *Rng* = 0.33–0.77, *W* = 48.0, *p* = 1.00, and *r*_*rb*_ < 0.01), this is illustrated in Figure 2.

Alzheimer’s disease subjects with anosognosia also displayed a lower GMD in the bilateral cingulum cortex ROI (*M* = 0.41, *Mdn* = 0.43, *IQR* = 0.09, and *Rng* = 0.34–0.48) than subjects without anosognosia (*M* = 0.43, *Mdn* = 0.45 *IQR* = 0.10, and *Rng* = 0.29–0.53), but this difference was not statistically significant (*W* = 39.0, *p* = 0.47, and *r*_*rb*_ = 0.24).

### White Matter Fractional Anisotropy Comparisons

No statistically significant differences in hippocampal FA were found between aMCI subjects with anosognosia (*M* = 0.29, *Mdn* = 0.31 *IQR* = 0.10, and *Rng* = 0.21–0.37) and without anosognosia (*M* = 0.29, *Mdn* = 0.27, *IQR* = 0.10, *Rng* = 0.20–0.37, *W* = 33.5, *p* = 0.85, and *r*_*rb*_ = −0.07). This was also the case in the cingulum FA, with no significant differences between aMCI with anosognosia (*M* = 0.44, *Mdn* = 0.47 *IQR* = 0.06, and *Rng* = 0.36–0.53) and without anosognosia (*M* = 0.44, *Mdn* = 0.42 *IQR* = 0.09, *Rng* = 0.36–0.53, *W* = 31.5, *p* = 0.71, and *r*_*rb*_ = −0.13).

Subjects with AD had similar results, with no significant differences in hippocampal FA between the anosognosia (*M* = 0.25, *Mdn* = 0.25, *IQR* = 0.11, and *Rng* = 0.15–0.39) and no anosognosia groups (*M* = 0.25, *Mdn* = 0.24 *IQR* = 0.04, *Rng* = 0.16–0.32 *W* = 28.0, *p* = 0.91, and *r*_*rb*_ = 0.01), and also no significant differences in cingulum FA between the groups (*anosognosia group M* = 0.38, *Mdn* = 0.36, *IQR* = 0.06, and *Rng* = 0.30–0.52; *non-anosognosia group M* = 0.41, *Mdn* = 0.40, *IQR* = 0.05, *Rng* = 0.34–0.49, *W* = 39.0, *p* = 0.23, and *r*_*rb*_ = 0.39).

### White Matter Mean Diffusivity Comparisons

No statistically significant differences were found in hippocampal MD between aMCI subjects with anosognosia (*M* = 7.31e−4, *Mdn* = 5.07e−4, *IQR* = 4.71e−4, and *Rng* = 4.47e−4 – 8.07e−4) and without anosognosia (*M* = 7.25e−4, *Mdn* = 0.27, *IQR* = 0.10, *Rng* = 0.20–0.37, *W* = 30.0, *p* = 0.62, and *r*_*rb*_ = −0.17). This was also the case in the cingulum MD, with no significant differences between aMCI with anosognosia (*M* = 5.98e−4, *Mdn* = 4.97e−4 *IQR* = 2.43e−4, and *Rng* = 4.52e−4 – 8.39e−4) and without anosognosia (*M* = 6.28e−4, *Mdn* = 6.35e−4 *IQR* = 3.05e−4, *Rng* = 4.47e−4 – 8.07e−4, *W* = 32.5, *p* = 0.78, and *r*_*rb*_ = −0.10).

In the AD group, no significant differences were found in hippocampal MD between the anosognosia (*M* = 8.53e−4, *Mdn* = 6.32e−4, *IQR* = 7.00e−4, and *Rng* = 4.36e−4 - 1.00e−3) and no anosognosia groups (*M* = 9.67e−4, *Mdn* = 9.56e−4 *IQR* = 2.39e−4, *Rng* = 5.14e−4 - 2.00e−3 *W* = 29.0, *p* = 0.96, and *r*_*rb*_ = 0.04), and also no significant differences in cingulum MD between the groups (*anosognosia group M* = 6.81e−4, *Mdn* = 5.83e−4, *IQR* = 3.47e−4, and *Rng* = 4.63e−4 – 9.10e−4; *non-anosognosia group M* = 7.34e−4, *Mdn* = 7.91e−4, *IQR* = 1.28e−4, *Rng* = 4.78e−4 – 8.53e−4, *W* = 26.0, *p* = 0.86, and *r*_*rb*_ = −0.07).

## Discussion

One of the main findings in this study, where we assessed volumetric and white matter tract changes associated with anosognosia, is that a group of aMCI subjects displaying anosognosia had a lower hippocampal volume than a group of aMCI subjects without anosognosia. These groups did not differ significantly by age, sex distribution, MMSE score, or ICV, which suggests that these groups were comparable. However, even more interestingly, aMCI subjects with anosognosia had a similar hippocampal volume to subjects with AD in our study. In the following, we will discuss the implications in the clinical characterization of aMCI and anosognosia assessment, neural and cognitive mechanisms underlying anosognosia, limitations of our study, and suggest future lines of research.

First, taking a clinical perspective, the association of reduced hippocampal volume with the presence of anosognosia in aMCI is in line with previous studies that place anosognosia as a risk factor for progression to AD dementia (Spalletta et al., 2014; Scherling et al., 2016). Hippocampal atrophy is independently and strongly correlated to AD progression from its early stages and to a heightened risk of progression from aMCI to AD dementia (Izzo et al., 2020; Zhuo et al., 2021). This implies that in clinical practice, an aMCI patient presenting to consultation with anosognosia might have a higher risk of progressing to dementia due to AD, and could benefit from a closer follow-up, a more comprehensive diagnostic workup, and potentially, disease-modifying strategies. Interestingly, our findings were obtained through a straight-forward classification of anosognosia. By using the screening questions of the BDSI (Godefroy et al., 2010), which are presented in Table 1, we dichotomized subjects as presenting and not presenting anosognosia, which is a simple approach that could be easily undertaken in everyday clinical assessments.

Regarding the neural and cognitive mechanisms underlying anosognosia, it has been hypothesized that because subjects affected by AD suffer from an inability to form new memories, they depend on remote personal semantics to evaluate their present performance, with inadequate self-appraisal (Morris and Mograbi, 2013; Vannini et al., 2017; Tondelli et al., 2018). This might explain why in our study anosognosia was significantly related to localized volumetric changes in the hippocampus, a fundamental structure for the formation of episodic memories (Eichenbaum, 2017). On the other hand, it is worth noting that AD subjects with anosognosia also displayed lower hippocampal and cingulum cortex volumes than AD subjects without anosognosia, but this difference was not statistically significant. This might imply that anosognosia in aMCI is an appropriate clinical marker of a brain phenotype that is within the AD continuum, but that anosognosia in AD arises from more subtle brain changes not limited to the hippocampus.

Some limitations of the current study are worth commenting on. The small sample size warrants caution in the interpretation of the results, which are thus in need of replication in larger samples. In addition, this implies that more subtle brain changes related to anosognosia might not have been detected. Another limitation that might explain the lack of differences in white matter tracts in our sample, is the concern that diffusion methods such as FA might be insufficient to study structures such as the cingulum bundle, where crossing-fiber anatomy might necessitate more specific measurements that track white matter-fibers more reliably (Douaud et al., 2011; Jeurissen et al., 2014).

Our findings are in contrast to recent studies that identify a link between poor awareness of memory performance and a loss of white-matter integrity in the corpus callosum, frontal-striatal fibers and anterior thalamocortical radiations, as well as in the full right hemisphere (Bertrand et al., 2021; Chang et al., 2021). Beyond the fact that the number of ROIs used in the current study is limited by a small sample size, it is worth noting that the definition of anosognosia used in each study (different assessments of awareness of memory performance vs. structured questionnaire of everyday behavior) limits the comparability of the findings.

In sum, our preliminary findings, if replicated, suggest that anosognosia might be a relevant clinical marker for the suspicion of structural brain changes within the AD continuum in subjects with aMCI. Future studies assessing larger populations are necessary in order to contribute both to the characterization of aMCI subtypes and the understanding of the neural changes underlying anosognosia.

## Data Availability Statement

The raw data supporting the conclusions of this article will be made available by the authors, without undue reservation.

## Ethics Statement

The studies involving human participants were reviewed and approved by National Institute of Neurology and Neurosurgery Mexico City. The patients/participants provided their written informed consent to participate in this study.

## Author Contributions

JF-V: study design, data collection, statistical design, data processing and analysis, and drafting of the manuscript. GR-G: data processing and analysis, and drafting of the manuscript. OM-M: study design, data collection, and the manuscript revision. RA-L: statistical design and manuscript revision. ML: study design, statistical design, and the manuscript revision. YR-A: study design and data collection. GA-C: study design and data analysis. RR: study design and the manuscript revision. AA: study design, acquisition of funding, and the manuscript revision. SE-G: theoretical background, study design, and the manuscript revision. AS-O: theoretical background, study design, acquisition of funding, and drafting of the manuscript. All authors contributed to the article and approved the submitted version.

## Conflict of Interest

The authors declare that the research was conducted in the absence of any commercial or financial relationships that could be construed as a potential conflict of interest.

## Publisher’s Note

All claims expressed in this article are solely those of the authors and do not necessarily represent those of their affiliated organizations, or those of the publisher, the editors and the reviewers. Any product that may be evaluated in this article, or claim that may be made by its manufacturer, is not guaranteed or endorsed by the publisher.
