# Supplementary material for: Anosognosia in Amnestic Mild Cognitive Impairment Is Related to Diminished Hippocampal Volume Comparable to Alzheimer’s Disease Dementia: Preliminary MRI Findings
Source: Front Aging Neurosci. 2021 Oct 28;13:739422. doi: 10.3389/fnagi.2021.739422 (PMC8581404; doi:10.3389/fnagi.2021.739422)
Supplement: Supplementary file 1 [file Data_Sheet_1.docx]

**SUPPLEMENTARY MATERIAL**

In an exploratory analysis, we assessed the relationship between the frequency and severity of anosognosia in aMCI and AD participants and MRI measurements (GMD, FA and MD). To this end, the product of frequency times severity obtained on the BDSI was calculated (see table 1) and this was correlated with each MRI measurement in the grey matter and white matter ROIs using Spearman’s correlation test. The results of these analyses are described below and are illustrated in figure S1.

In aMCI participants, hippocampal GMD was moderately and correlated with the frequency-severity anosognosia score, this relationship was statistically significant (*ρ* = -0.54, *p* = 0.02). On the other hand, the cingulum GMD was not significantly correlated with the anosognosia score (*ρ* = -0.26, *p* = 0.31). In AD participants, neither hippocampal GMD (*ρ* = -0.29, *p* = 0.27) or cingulum GMD (*ρ* = -0.22, *p* = 0.43) were significantly correlated with the anosognosia score.

In aMCI participants, anosognosia scores were not significantly correlated with hippocampal (*ρ* = 0.03, *p* = 0.92) or cingulum FA (*ρ* = 0.09, *p* = 0.72). This was also the case with AD participants, where the correlations between anosognosia score and hippocampal (*ρ* = -0.16, *p* = 0.56) or cingulum FA (*ρ* = -0.46, *p* = 0.08) were not significant.

Regarding MD correlations with anosognosia scores, these were not significant in aMCI participants in either the hippocampal (*ρ* = 0.19, *p* = 0.43) or cingulum (*ρ* = 0.13, *p* = 0.61) ROI. This was also the case in AD participants (hippocampal MD-anosognosia score: *ρ* = 0.13, *p* = 0.65; cingulum MD-anosognosia score *ρ* = 0.20, *p* = 0.47).

Although most of these correlations were not significant, there are trends shown in figure S1 that could inform future research, particularly the negative correlation between anosognosia scores and FA in both ROIs shown in AD participants. If replicated, this suggests that white-matter integrity disturbances could result in a more pronounced anosognosia presentation in AD.


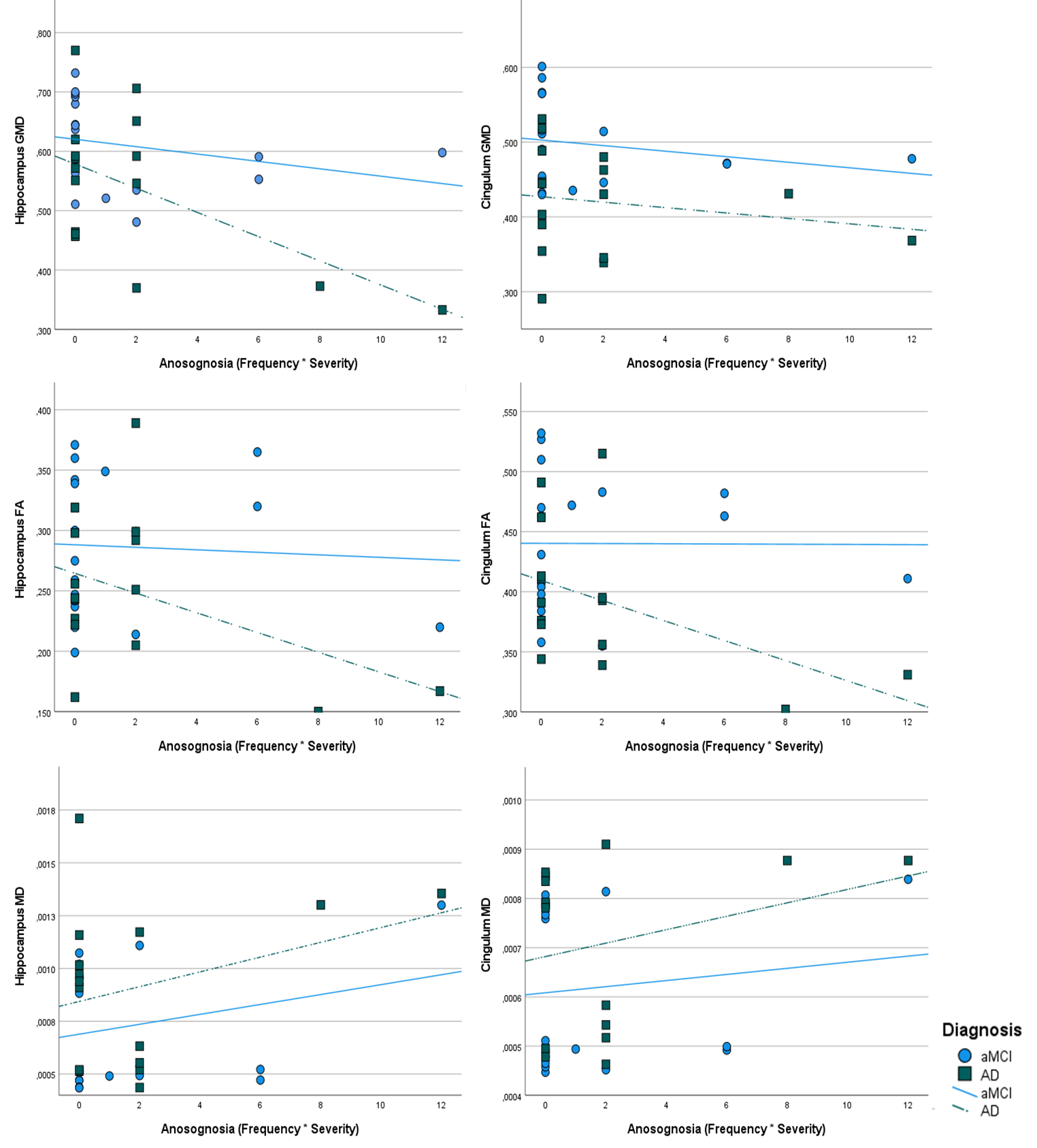


**Figure S1:** Correlations between anosognosia scores and MRI metrics. Only the correlation between hippocampal GMD was significantly correlated with the anosognosia score.

aMCI: amnestic mild cognitive impairment; AD: Alzheimer’s disease. GMD: Gray matter density, FA: Fractional anisotropy; MD: Mean diffusivity; aMCI: Amnestic mild cognitive impairment, AD: Alzheimer's disease.
